# The Principles of Bacteriology

**Published:** 1896-08

**Authors:** 


The Principles of Bacteriology. A Practical Manual for Students and Physicians. By A. C. Abbott, M. D., First Assistant Laboratory of Hygiene, University of Pennsylvania, Philadelphia. Third edition, enlarged and thoroughly revised. Duodecimo, 492 pages, with ninety-eight illustrations, of which seventeen are colored. Cloth, $2.50. Philadelphia: Lea Brothers & Co., Publishers.

The second edition of this popular little work was reviewed by the writer in the January, 1895, number of the Journal, page 380, and its many good points pointed out. The appearance of another edition in such a short time demonstrates fully its acceptability and, therefore, desirability by students and the profession, and the author is to be congratulated on his efforts. The third edition is subject to a few changes over the second, mainly, however, in keeping pace with the rapid strides made in bacteriology.

